# The Bisindole Alkaloid Caulerpin, from Seaweeds of the Genus *Caulerpa*, Attenuated Colon Damage in Murine Colitis Model

**DOI:** 10.3390/md16090318

**Published:** 2018-09-07

**Authors:** Alessandra M. M. Lucena, Cássio R. M. Souza, Jéssica T. Jales, Paulo M. M. Guedes, George E. C. de Miranda, Adolpho M. A. de Moura, João X. Araújo-Júnior, George J. Nascimento, Kátia C. Scortecci, Barbara V. O. Santos, Janeusa T. Souto

**Affiliations:** 1 Department of Microbiology and Parasitology, Federal University of Rio Grande do Norte, Avenida Salgado Filho, BR 101, Campus Universitario, Lagoa Nova, Natal, RN 59078-900, Brazil; ale.marinho@hotmail.com (A.M.M.L.); cassiormsouza@gmail.com (C.R.M.S.); jessica_jales@hotmail.com (J.T.J.); guedespmm@gmail.com (P.M.M.G.); 2 Laboratory of Marine Algae, Department of Systematics and Ecology, Federal University of Paraiba, Joao Pessoa, PB 58051-900, Brazil; mirandag@dse.ufpb.br; 3 Laboratory of Animal Experimentation of the Institute of Immunobiological Technology (LAEAN/Bio-Manguinhos), Rio de Janeiro, RJ 21040-900, Brazil; adolpho.moura@bio.fiocruz.br; 4 School of Nursing and Pharmacy, Federal University of Alagoas, Maceió, AL 57072-900, Brazil; jotaaraujo2004@gmail.com; 5 Academic Unit of Biological Sciences, Federal University of Campina Grande, Patos, PB 58708-110, Brazil; geonascimento79@yahoo.com.br; 6 Department of Cell Biology and Genetics, Federal University of Rio Grande do Norte, Avenida Salgado Filho, BR 101, Campus Universitario, Lagoa Nova, Natal, RN 59078-900, Brazil; kacscort@yahoo.com; 7 Postgraduate Program in Natural Products and Synthetic Bioactive and Department of Pharmaceutical Sciences, Federal University of Paraíba, João Pessoa, PB 58051-900, Brazil; barbara@ltf.ufpb.br

**Keywords:** caulerpin, ulcerative colitis, anti-inflammatory activity, cytokines, tissue damage

## Abstract

Caulerpin (CLP), an alkaloid from algae of the genus Caulerpa, has shown anti-inflammatory activity. Therefore, this study aimed to analyze the effect of CLP in the murine model of peritonitis and ulcerative colitis. Firstly, the mice were submitted to peritonitis to evaluate which dose of CLP (40, 4, or 0.4 mg/kg) could decrease the inflammatory infiltration in the peritoneum. The most effective doses were 40 and 4 mg/kg. Then, C57BL/6 mice were submitted to colitis development with 3% dextran sulfate sodium (DSS) and treated with CLP at doses of 40 and 4 mg/kg. The disease development was analyzed through the disease activity index (DAI); furthermore, colonic tissue samples were submitted to histological analysis, NFκB determination, and in vitro culture for cytokines assay. Therefore, CLP at 4 mg/kg presented the best results, triggering improvement of DAI and attenuating the colon shortening and damage. This dose was able to reduce the TNF-α, IFN-γ, IL-6, IL-17, and NFκB p65 levels, and increased the levels of IL-10 in the colon tissue. Thus, CLP mice treatment at a dose of 4 mg/kg showed promising results in ameliorating the damage observed in the ulcerative colitis.

## 1. Introduction

The mammalian intestinal surface is in continuous contact with the external environment and, consequently, is in constant interaction with a wide variety of microorganisms (pathogenic or commensals) and food antigens [1]. Microorganisms from the intestinal microbiota are responsible for the maintenance of the immune tolerance of the bowel by sending signals to the epithelial cells. Those signals induce the production of molecules, especially TGF-β and IL-10, which lead to inhibition of the immune response against these microorganisms [2]. The injury to the intestinal epithelium, also known as dysbiosis, leads to the breakdown of tolerance and homeostasis of the intestinal immune system [2]. This situation causes an inflammatory response, with an imbalance between the populations of inflammatory T helper cells (Th1, Th2, and Th17) and regulatory T lymphocytes.

The recurrent and chronic inflammation of the intestinal mucosa leads to the emergence of Inflammatory Bowel Diseases (IBD), such as ulcerative colitis. These diseases lead to clinical signs and symptoms, such as diarrhea with blood and/or mucus, weight loss, increased bowel movements, nausea, vomiting, and abdominal pain [3,4]. Ulcerative colitis is generally related to the cecum, colon, and rectum regions, and its histological changes are limited to the mucosa [3,4]. Such inflammatory diseases generally do not decrease the life expectancy of patients but lead to a poor quality of life due to their signs and symptoms [4], besides causing a predisposition to the development of intestinal neoplasia [5]. It has been described that in some situations of ulcerative colitis, patients may develop peritonitis, especially in more severe cases, with abscesses or intestinal perforation, due to the severity of the inflammatory condition, requiring surgical intervention to heal the condition [6]; this process seems to be associated with a persistent neutrophil infiltrate that leads to reduced epithelial barrier function in the gut, causing the tissue damage observed [7].

The treatment of these diseases aims to solve or reduce the inflammatory process, improve the patients’ quality of life by ameliorating symptoms, prevent the emergence of tumors, and decrease the requirement for surgical intervention [8,9]. The treatment is based on the localization, severity, and extent of inflammation [4,9]. A diversity of agents such as aminoglycosides, glucocorticoids, immunosuppressive agents, and monoclonal antibodies is used [9], but their use has generated the occurrence of several important adverse effects [9,10]. In mice, a model for studying ulcerative colitis is inducing intestinal inflammation through a solution of 3% dextran sulfate sodium (DSS) in the drinking water of the animals.

Considering the severity and the threatening consequences of IBD in addition to the adverse effects of traditional treatments, it is necessary to study alternative therapies to treat individuals affected by these diseases. 

The ocean shelters most of the global biodiversity that makes the marine environment a great reservoir of bioactive natural products that are mostly not found in the terrestrial environment. Seaweeds have been shown to be a great source of bioactive natural products, such as antineoplastic [11], anti-inflammatory [12], antinociceptive [13], anticoagulant, antithrombotic [14], antimicrobial and antioxidative [15], antidiabetic [16], anti-aging, and anti-hyperpigmentation activities [17].

**Figure 1:**
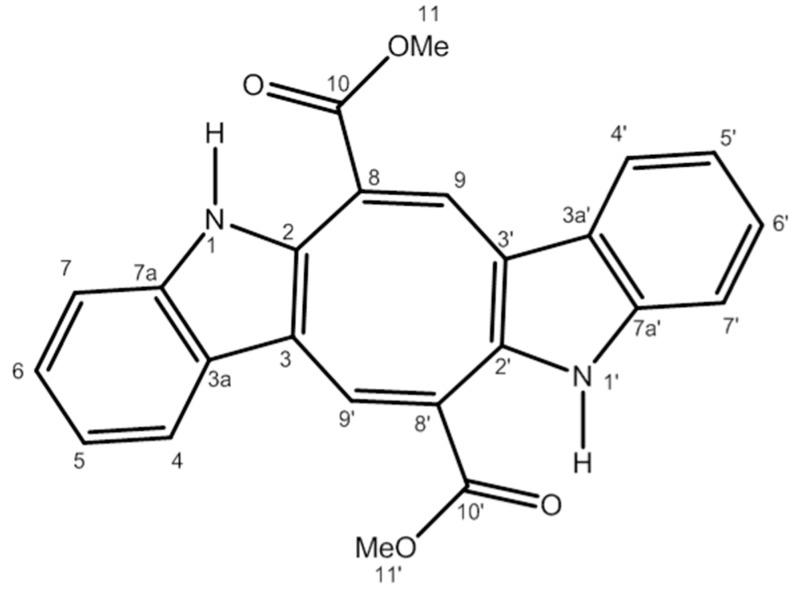
The chemical structure of caulerpin.

Extracts of algae of the genus Caulerpa and their purified products have shown a protective effect in models of inflammation. Extracts of algae *Caulerpa sertularioide* and *Caulerpa mexicana* showed anti-inflammatory activity by significantly inhibiting leukocyte migration into the peritoneal cavity in an animal model of peritonitis induced by carrageenan [13]. Species of the genus Caulerpa have many chemical compounds, such as alkaloids, steroids, phenylpropanoids, lipids, and indole terpenes, among which is the bisindole alkaloid caulerpin (CLP) (Figure 1) [18]. It is present in 80% of the species of algae of the genus Caulerpa; however, it can be found in other algae genera [19]. Diverse biological activities of CLP have been described, including antinociceptive, anti-inflammatory [20], antiviral [21], antituberculosis [22], antineoplastic [23], and antispasmodic [24]. Similar to CLP, other bisindole alkaloids have been shown to have anti-inflammatory activity in different parameters of inflammation and have presented different mechanisms of action, ranging from inhibition of the COX-2 pathway to inhibition of the NFκB and MAP kinase pathways and increased expression of adenosine A2A receptors (A2AR) that have anti-inflammatory activity [25].

Therefore, alternative therapies for IBD are needed, and based on the diverse biological activities presented by CLP, especially the anti-inflammatory activity, further study is needed on the action of the substance as a therapeutic agent in a murine model of colitis induced by DSS. Moreover, the refinement of the knowledge concerning this alkaloid present in many algae of the genus Caulerpa is needed, with the aim of expanding the use of natural products in the treatment of different inflammatory diseases. In this way, the aim of our study was to evaluate the effects of CLP on cell migration into the peritoneal cavity in animals stimulated with zymosan, in the time of 6 h, which is preferentially when there is a polymorphonuclear infiltrate [26], as well as evaluate its effects in a model of colitis, in which neutrophils play an important role in this pathogenesis as already mentioned.

## 2. Results

### 2.1. Caulerpin Decreases Cell Migration to Peritoneal Cavity in Model of Peritonitis Induced by Zymosan

**Figure 2:**
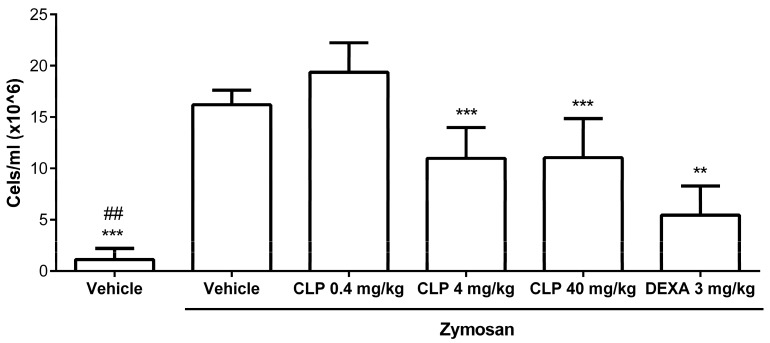
Effect of different doses of caulerpin (CLP) on the cell migration to the peritoneal cavity after zymosan injection. Mice were treated orally with CLP at doses of 40, 4, and 0.4 mg/kg and dexamethasone (DEXA) at the dose of 3 mg/kg, 30 min before being injected intraperitoneally with zymosan (40 mg/kg). Each point represents the mean ± SD from the tested animals. The data are representative of three independent experiments, with five animals per group in each experiment (N = 5). ## *p* < 0.05, vehicle versus zymosan + vehicle. ** *p* < 0.01 and *** *p* < 0.001, zymosan + vehicle versus zymosan + CLP 4 and 40 mg/kg or zymosan + DEXA.

Firstly, to evaluate the best dose of CLP that could have anti-inflammatory activity in vivo, we performed a zymosan-induced peritonitis model and assessed cell migration into the peritoneal cavity after the zymosan stimulation and CLP treatment. Amongst the three doses of CLP tested, the 40 and 4 mg/kg doses showed the best results in inhibiting cell migration into the peritoneal cavity of animals injected with zymozan compared to the group that received the vehicle by oral route (o.r.) + zymosan intraperitoneally (i.p.), called the vehicle + zymosan group (*p* < 0.001). The 0.4 mg/kg dose did not obtain significant reduction. The reference anti-inflammatory drug, dexamethasone (DEXA), significantly inhibited cell migration into the peritoneal cavity of the mice receiving zymosan i.p. (Figure 2), when compared to the vehicle + zymosan group (*p* < 0.001) and did not present significant difference when compared to the results of CLP at the doses of 40 and 4 mg/kg. Thus, CLP at the doses of 40 and 4 mg/kg were chosen for the treatment of mice in a murine model of colitis induced by 3% DSS.

### 2.2. The Treatment of Mice with Caulerpin Ameliorates the Body Weight Loss and the Disease Activity Index Induced by DSS

**Figure 3:**
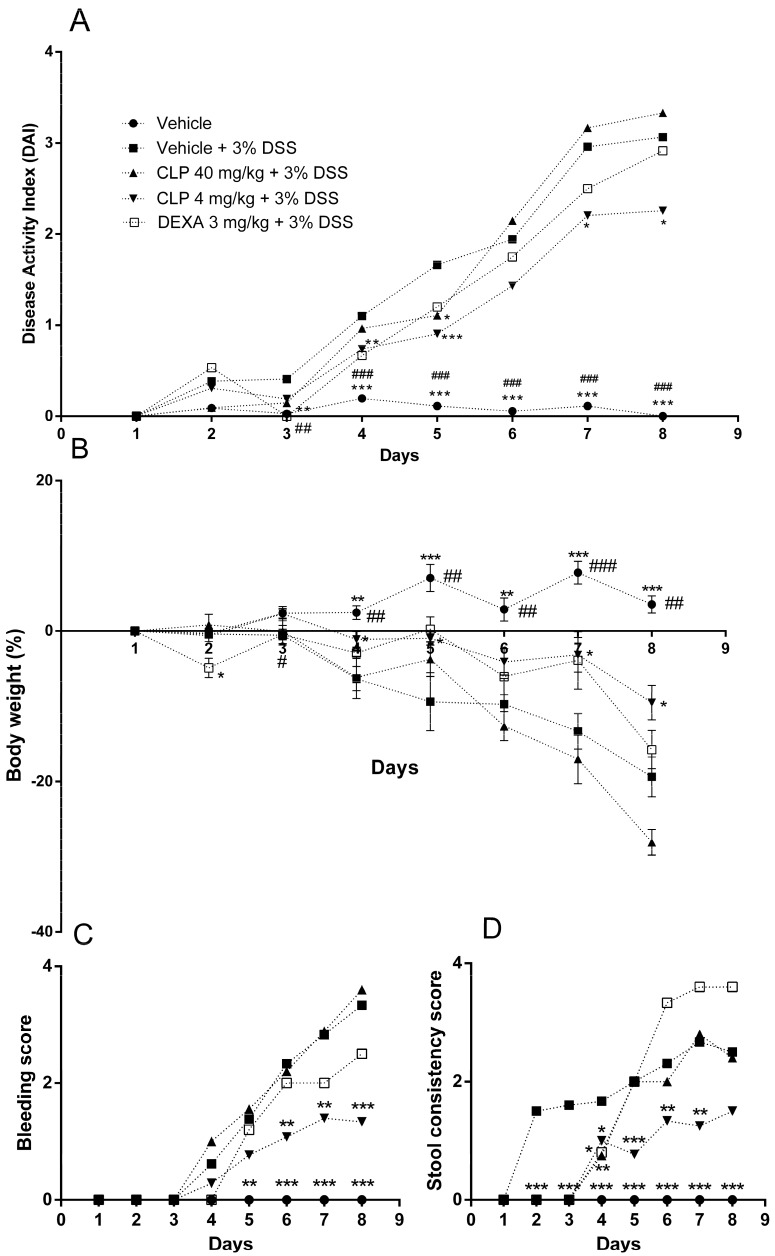
Effect of caulerpin (CLP) treatment on the Disease Activity Index (DAI) after DSS-colitis induction. The development of colitis was assessed by the DAI, which considers animal weight loss, presence of blood in the stool, and stool consistency. The data are representative of three independent experiments. (**A**) Disease activity index, (**B**) body weight (%), (**C**) bleeding score, and (**D**) stool consistency score. Each point represents the mean ± SD from the tested animals. The data are representative of three independent experiments, with five animals per group in each experiment (N = 5). ### *p* < 0.001, ## *p* < 0.01 and # *p* < 0.05, vehicle + water versus vehicle + 3% DSS. *** *p* < 0.001, ** *p* < 0.01 and * *p* < 0.5, vehicle + 3% DSS versus CLP 4 mg/kg + 3% DSS.

The next step was to evaluate the effect of CLP treatment on the murine model of colitis induced by 3% dextran sulfate sodium (DSS) solution. The animals that received only 3% DSS in the drinking water and were treated with the vehicle by oral route (called vehicle + 3% DSS group) showed significant clinical signs (Figure 3A) of ulcerative colitis development after the third day of DSS administration with progressive weight loss after the fourth day of disease induction (Figure 3B). Stool softened after the second day (Figure 3C) with presence of blood after day four (Figure 3D). On the other hand, the treatment of mice with CLP at the dose of 4 mg/kg led to attenuation of clinical signs of experimental colitis (Figure 3A) by delaying body weight loss until the sixth day (Figure 3B) and the onset of clinical signs until the fourth day (Figure 3C,D). However, the treatment of mice with the dose of 40 mg/kg of CLP and dexamethasone (DEXA) did not show protective effects. 

### 2.3. The Treatment of Mice with Caulerpin Attenuates the Colon Tissue Damage Induced by DSS

**Figure 4:**
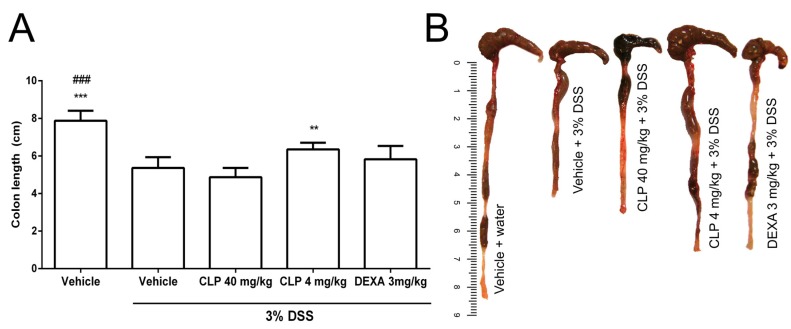
Effect of caulerpin (CLP) treatment on the colon length in DSS-induction colitis. At the end of induction of the experimental colitis, the colons of the animals were removed and measured. The data are representative of three independent experiments. Each point represents the mean ± SD from the tested animals. The data are representative of three independent experiments, with five animals per group in each experiment (N = 5). ### *p* < 0.05, vehicle versus vehicle + 3% DSS. *** *p* < 0.001 and ** *p* < 0.01, vehicle + 3% DSS versus CLP 4 mg/kg.

The vehicle + 3% DSS group showed shortening of the colon compared to the vehicle + water group, demonstrating the development of ulcerative colitis (Figure 4A,B). The treatment of animals with 4 mg/kg of CLP (CLP 4 mg/kg + DS3% S group) reduced the shortening of the colon length when compared with the vehicle + 3% DSS group or groups treated with CLP at the dose of 40 mg/kg or dexamethasone (DEXA) (Figure 4).

**Figure 5:**
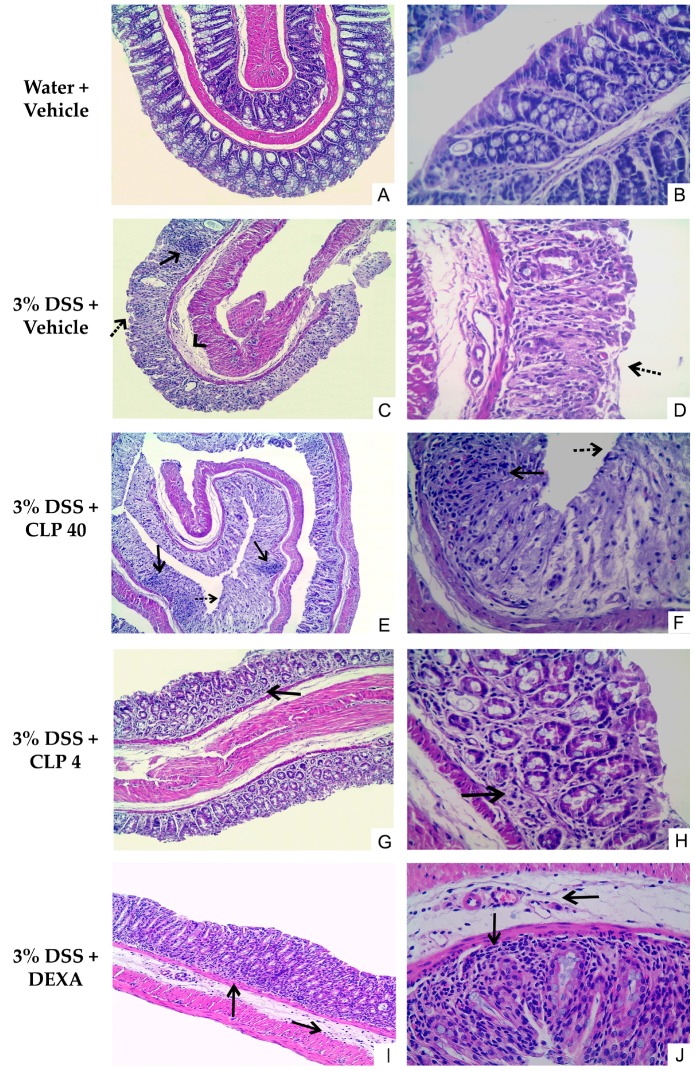
Effect of caulerpin (CLP) treatment on the colon damage after DSS-induction colitis. Histological sections of colonic mucosa from DSS colitic mice stained with hematoxylin and eosin, showing the effects of treatment when administered. (**A**,**B**) water + vehicle; (**C**,**D**) DSS + vehicle; (**E**,**F**) CLP 40 mg/kg; (**G**,**H**) CLP 4 mg/kg; (**I**,**J**) DEXA 3 mg/kg. Original magnification in A, C, E, G, and I was 100×; in B, D, F, H, and J, was 400×.

Histological analysis was performed on segments of the distal colon. In Figure 5, it can be observed that in the specimens of mice not submitted to colitis induction, the distal colon had normal morphology with preserved tissue architecture (Figure 5A,B). The opposite was observed in the animals exposed to 3% DSS, in which analysis of the histological sections revealed that the distal colon had moderate to severe lesions (Figure 5C,D). In these sections, it was possible to observe loss of parenchyma tissue, intense tissue ulceration (Figure 5C, black dotted arrow), glandular destruction with disappearance of Goblet cells, fibrosis, edema (Figure 5C, head of arrow), presence of an intense mononuclear inflammatory infiltrate (mainly lymphocytic), and neutrophilic abscesses (Figure 5C, arrow solid line) for almost the entire mucosal extension. In the animals treated with CLP at a dose of 40 mg/kg (Figure 5E,F), no improvement was observed in the histological damage caused by the administration of DSS, as already shown in the other aspects analyzed above (weight loss, DAI, and morphometric analysis). Thus, the same characteristics observed in the 3% DSS + vehicle group could be observed in the distal colon of the animals of this group, with evidence of sites of inflammatory infiltrate (Figure 5E, arrow solid line), loss of parenchyma structure, neutrophilic abscesses (Figure 5E, arrow solid line), and presence of ulcerations of the epithelium (Figure 5E, black dotted arrow). On the other hand, it was observed that the treatment of animals with CLP (4 mg/kg) and dexamethasone (DEXA) reduced colon tissue damage induced by DSS solution (Figure 5G–J). In the animals treated with CLP at a dose of 4 mg/kg, a slight improvement was observed with the preservation of tissue morphology, with a reduction in the number of mononuclear cells, which were predominantly lymphocytes, in the inflammatory infiltrate located in the lamina propria (Figure 5G, arrow solid line), and the presence of glands and Goblet cells, when compared to the tissue changes observed in the animals treated with 3% DSS + vehicle (Figure 5G,H). Similarly, in the group of animals treated with dexamethasone and receiving DSS, a discrete reduction in the distribution of the areas with inflammatory infiltrate, mainly lymphocytic, in the lamina propria and submucosa (Figure 5I, arrows solid line) was observed, as well as preservation of tissue morphology and maintenance of the glands and Goblet cells (Figure 5I,J).

### 2.4. The Treatment of Mice with Caulerpin Decreases the Production of Pro-Inflammatory Cytokines in DSS Colitis Model

**Figure 6:**
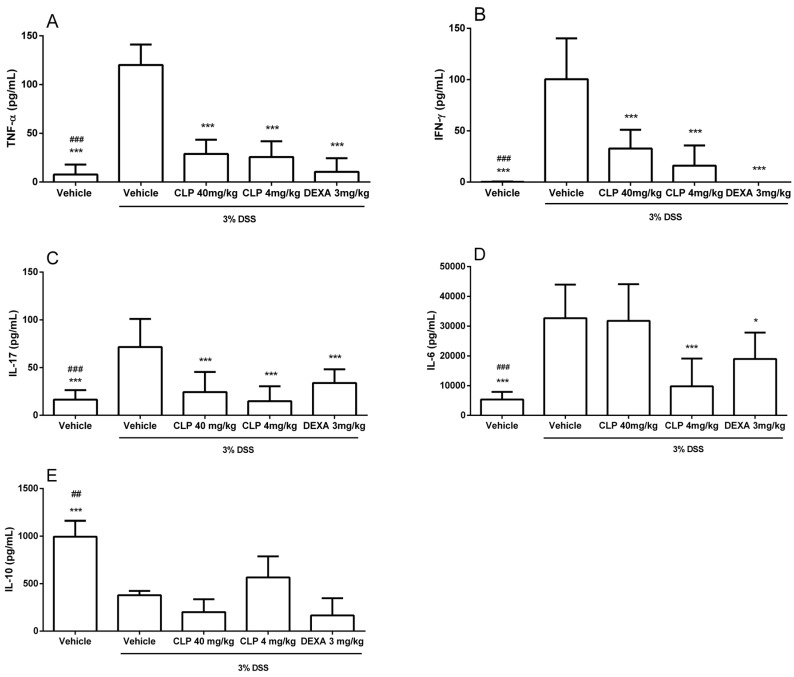
Effect of caulerpin treatment on the production of cytokines in colon culture supernatants in DSS-induced colitis. (**A**) TNF-α; (**B**) IFN-γ; (**C**) IL-17A; (**D**) IL-6; (**E**) IL-10. At the end of induction of experimental colitis, the tissue was washed, deposited in a 24-well plate containing culture medium, and incubated for 24 h, and the supernatants were collected and frozen for TNF-α, IFN-γ, IL-17A, IL-6, IL-10, and TGF-β1 levels measurement by enzyme-linked immunosorbent assay kits. These data are representative of three independent experiments. Each point represents the mean ± SD from the tested animals. The data are representative of three independent experiments, with five animals per group in each experiment (N = 5). ### *p* < 0.001 and ## *p* < 0.01, vehicle versus vehicle + 3% DSS. *** *p* < 0.001 and * *p* < 0.05, vehicle + 3% DSS versus treatment groups (DSS + CLP and DSS + DEXA).

The data presented in Figure 6 show an increase in the production of TNF-α (Figure 6A), IFN-γ (Figure 6B), IL-17 (Figure 6C), and IL-6 (Figure 6D), and a decrease in IL-10 (Figure 6E) in the colon culture supernatant of animals receiving 3% DSS in the drinking water (vehicle + 3% DSS) when compared with the vehicle group. However, those animals treated with CLP at doses of 40 and 4 mg/kg showed a reduction in the levels of pro-inflammatory cytokines, similar to that observed for animals treated with dexamethasone (DEXA—3 mg/kg), and an increase in IL-10 production when compared to animals receiving no treatment, with this difference better observed at the 4 mg/kg dose.

### 2.5. The Treatment of Mice with Caulerpin Decreases the Expression of NFkB p65 in DSS Colitis Model

**Figure 7:**
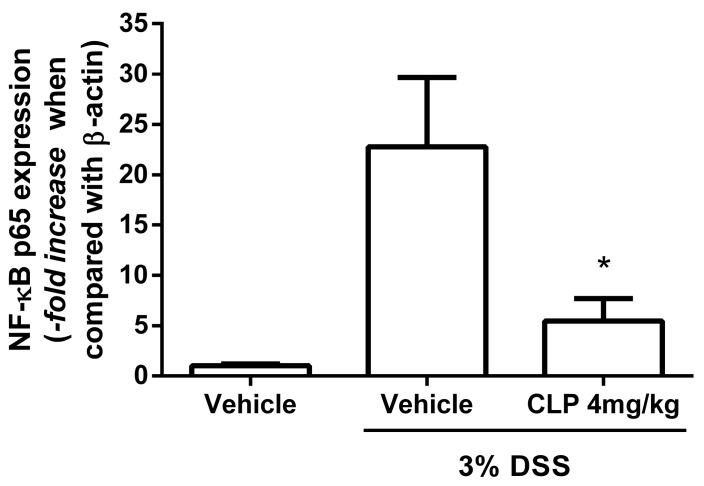
Effect of treatment with caulerpin on the expression of NF-κB p65 in the colon in the DSS-induced colitis model. The expression of the p65 subunit of NF-κB and β-actin, used as the reference gene for normalization, was performed by qPCR. The graph represents the expression level of NF-κB p65 from the vehicle + 3% DSS and the groups that received DSS and were treated with CLP (4 mg/kg) when compared with the group which received water + vehicle. Each point represents the mean ± SD from the tested animals. The data are representative of one independent experiment, with five animals per group in the experiment (N = 5). * *p* < 0.01, vehicle + 3% DSS versus CLP 4 mg/kg + 3% DSS.

Since the dose of CLP that showed the best anti-inflammatory effects in our DSS-induced colitis model was the dose of 4 mg/kg, our next step was to evaluate which mechanism of this alkaloid could be exerting its anti-inflammatory effects. For this, we evaluated the effect of CLP on the expression of NFκB, which is an important transcription factor involved in the expression of the pro-inflammatory cytokines investigated in this study. The data presented in Figure 7 show an increase in NF-κB p65 expression in the colon of animals submitted to the DSS-induced colitis model (Vehicle + DSS), corroborating with the increase in the pro-inflammatory cytokine production observed in Figure 6. The treatment with CLP at doses of 4 mg/kg (CLP 4 + DSS) was able to reduce the expression of this transcription factor when compared to levels of expression of vehicle + 3% DSS group. No statistical difference was observed between the group treated with CLP and the Vehicle group.

## 3. Discussion

The initial goal of this study was to evaluate the effect of CLP (structure in Figure 1) in a murine model of peritonitis, in order to determine the most effective dose of this alkaloid that would be able to inhibit cell migration to the peritoneal cavity of animals inoculated with zymosan. The experimental model of peritonitis allows the evaluation of the leukocyte cell migration, through leukocyte count, present in the liquid of the peritoneal cavity after administration of zymosan. In addition, in some severe cases of ulcerative colitis, peritonitis has been observed in patients due to abscesses and intestinal perforation, associated with a persistent neutrophilic infiltrate leading to reduced barrier function in the intestine, causing tissue damage with loss of fecal material in the peritoneum, which causes this peritonitis [6,7].

In our study, two of three doses of CLP used had good results in inhibiting cell migration into the peritoneal cavity of animals injected with zymozan, 40 and 4 mg/kg. These data are in accordance with our previous publication showing that aqueous and methanolic extracts of *Caulepa mexicana* exerted an anti-inflammatory effect, reducing zymosan-induced cell recruitment into the peritoneal cavity [27]. Indeed, these doses of CLP were tested to evaluate their antinociceptive effect [20] and it was found that the three doses evaluated had an antinociceptive effect, and the dose of 100 μmol/kg, which corresponds to our 40 mg/kg dose, had the best effect in this model. In the same study, the dose of 100 μmol/kg also showed anti-inflammatory activity in ear edema and peritonitis models. The authors suggest that the indole group of CLP is probably responsible for the antinociceptive and anti-inflammatory activities of this alkaloid and that the mechanisms of actions of this substance can involve the inhibition of COX and antioxidant activity [20].

The time of cell migration evaluated in the peritonitis model was 6 h after the inoculation of zymosan. At that time, migration of polymorphonuclear cells occurs preferentially [26] and since these cells are responsible for causing damage in the colon in cases of colitis [7], generating a peritonitis in patients who develop ulcerative colitis [6], we evaluated the doses that were most effective in exerting anti-inflammatory activity in the peritonitis model to treat the animals in the DSS-induced colitis model. Results similar to those in our study were found in the literature, in which the beginning of clinical signs occurred between the second and fourth day of exposure to DSS solution in C57BL/6 mice without treatment (vehicle + 3% DSS) [28,29]. On the other hand, the treatment of mice with CLP at the dose of 4 mg/kg led to attenuation of clinical signs of experimental colitis (Figure 3A) by delaying the weight loss appearance until the sixth day (Figure 3B) and the onset of clinical signs until the fourth day (Figure 3C,D). These data are in accordance with previous studies from our group that have shown that the methanolic extract of *C. mexicana* ameliorates the clinical signs in animals with ulcerative colitis induced by DSS [30]. Similar to our data, the treatment of animals with the indole alkaloid Sinomenine protected the lungs of the mice against the damage caused by lipopolysaccharide in the model of acute lung injury, and this protection, according to the authors, happened due to an increased adenosine A2A (A2AR) receptor signaling, which has anti-inflammatory activity [31]. 

However, the treatment of mice with the dose of 40 mg/kg of CLP and dexamethasone did not show protective effects. In this same way, a study showed that that oral treatment with a high dose of nicotine was not effective in the DSS-induced colitis model whereas a lower dose could reduce the damage caused in intestinal mucosa by DSS [32], in a remarkable dose-dependent effect called the “Inverted U” effect, similar to our data with the doses of 4 and 40 mg/kg of CLP. On the other hand, the dose of 40 mg/kg of CLP showed anti-inflammatory activity in the model of capsaicin-induced ear edema and carrageenan-induced peritonitis [20]. These different findings may indicate that caulerpin could act differently depending on the dose at different sites of inflammation. Therefore, more studies need to be performed with this alkaloid to find its ligand in the cells and all its mechanisms of action. Although glucocorticoids, such as dexamethasone, have been used as reference anti-inflammatory treatment in animal models of IBD, with the expected benefit in terms of intestinal anti-inflammatory activity, this positive effect was not observed in the present study. Other studies using glucocorticoids as treatment control have observed the worsening of the general condition of the animal in the DSS IBD model [33,34]. Other natural compounds studied did not present better results when compared to dexamethasone in attenuation of clinical symptoms or evolution of DAI and weight loss in the treatment of DSS-induced ulcerative colitis in mice [35,36].

Once we observed that the CLP (4 mg/kg) ameliorated the weight loss and clinical signs in the murine model of ulcerative colitis, the next step was to evaluate the effect of this alkaloid in the colon tissue damage. The shortening of the colon of animals treated with DSS solution is taken as an indirect marker of the inflammatory process [37] and several studies related to the development of colitis have used this data as one of the evaluation parameters [29,37,38].

The data related to CLP (4 mg/kg) corroborate the results of clinical signs (Figure 3) and morphometric analysis (Figure 4) of the colon of animals in this group. However, demonstration of tissue repair is not often necessary to determine the clinical remission of patients; rather, it is important in reducing the risk of dysplasia in these individuals [9]. It shows that the CLP (4 mg/kg) could be a treatment that can help to minimize the risk of neoplasia. Although tissue damage was reduced in the dexamethasone group (Figure 5I,J), this treatment did not change the shortening of colon length caused by DSS. A previous study using the DSS IBD model and glucocorticoids had suggested the role of glucocorticoids in weakening the mucosal barrier by modulating epithelial dynamics, inhibiting cell proliferation, and slowing wound healing [39].

These data, in conjunction with clinical signs presented, point to CLP (4 mg/kg) as a mitigating agent in the inflammatory process triggered by the DSS solution. This effect in ameliorating the clinical signs of the experimental model may likely be due to the inhibitory action of CLP on the production of all tested pro-inflammatory intestinal cytokines—TNF-α, IFN-γ, IL-6, and IL-17, discussed below. In fact, nicotine, also an alkaloid, was used in low dose orally in the DSS colitis model, with improvement in clinical signs and reduction in the level of TNF-α [32]. Alkaloids of the plant *Sophora alopecuroides* also promoted a protective effect on animals submitted to the DSS chronic colitis model, and a possible inhibitory effect on NF-kB, an important transcription factor in the production of pro-inflammatory cytokines, was proposed [40]. Also, indolic alkaloids, such as Sinimenine and Oxymatrine, have shown protective effects in ameliorating tissue damage observed in models of acute lung injury and mastitis induced by LPS, associated with a decrease in neutrophil infiltrate, downregulation in the activation of NF-κB and MAPKs signal pathways [25,41], and upregulation of the A2A adenosine receptor (A2AR) [31].

As pro-inflammatory cytokines have been shown to play an important role in the development of ulcerative colitis in both human and murine models [4,42,43,44,45,46], our next step was to determine Th1 (IFN-γ and TNF-α), Th17 (IL-6 and IL-17), and Treg (IL-10) cytokine levels in the supernatant of the colon tissue culture from animals used in this study. The data presented in Figure 6 show an increase in the production of TNF-α (Figure 6A), IFN-γ (Figure 6B), IL-17 (Figure 6C), IL-6 (Figure 6D), and IL-10 (Figure 6E) in the colon culture supernatant of animals receiving 3% DSS in the drinking water. However, those animals treated with CLP at doses of 40 and 4 mg/kg showed a reduction in the levels of pro-inflammatory cytokines, similar to that observed for animals treated with dexamethasone (3 mg/kg), and an increase in IL-10 production when compared to animals receiving no treatment, with this difference better observed at the 4 mg/kg dose. Studies have shown that in ulcerative colitis, cytokines of the Th1 and Th17 patterns play an important role in the pathology of the disease [42,45,47], while Treg-like cytokines, such as IL-10, counterbalance the inflammatory process, reducing tissue damage [48,49]. A study with individuals affected by IBD showed high levels of IL-6, IL-1β, and TNF-α mRNA in their mucous membranes [50]. This study also described that the levels of IL-6 mRNA were most correlated with the degree of inflammation in the intestinal mucosa and this was expressed only in the inflamed mucosa—suggesting that IL-6 is a possible target in the IBD treatment. The present study demonstrated that CLP (4 mg/kg) was able to promote the reduction of these cytokine levels (Figure 6D). Increased levels of IL-17A seem to be linked to high levels of IL-6, since in the T_H_17 cell polarization the action of this cytokine is needed in conjunction with the TGF-β that is normally present in the intestine. IL-17 promotes the activation of NF-kB and MAPK pathways [51], which leads to the production of chemokines to increase the recruitment of neutrophils and T cells to the lamina propia [42]. Thus, reduction in IL-17 provided by CLP was favorable to the attenuation of intestinal mucosal lymphocytic and neutrophilic inflammation observed by clinical signs and morphological and histological analysis. Similar to our data, the treatment with the indole alkaloid oxymatrine significantly ameliorated the histopathologic changes in the murine model of mastitis, with a decrease in neutrophilic tissue infiltration, and in the production of pro-inflammatory cytokines TNF-α, IL-1β, and IL-6 in the mammary gland tissues, which was possibly linked with the inhibition of the activation of NF-κB and MAPKs signal pathways [41].

Thus, our data show a protective role of CLP in the model of ulcerative colitis, since the treatment of the animals with this alkaloid negatively regulated the levels of Th1 and Th17 cytokines and increased the production of IL-10, known as a cytokine with anti-inflammatory activity [52,53]. In agreement with these findings, we have recently shown that treatment of mice with the methanolic extract of *C. mexicana* significantly reduced tissue damage observed in the model of ulcerative colitis induced by DSS and this was associated with a dramatic decrease in the Th1 and Th17 cytokine production [30]. Thus, CLP, as well as the methanolic extract of *C. mexicana*, is protective against the damage induced by DSS in colon tissue, negatively regulating the production of pro-inflammatory cytokines and increasing anti-inflammatory cytokine levels. We also show that the treatment with caulerpin was able to decrease NF-κB p65 expression, which is important for the production of these cytokines. Therefore, this alkaloid could be acting to regulate this network of cytokines downregulating this important pro-inflammatory transcription factor, as shown previously in the colitis model for alkaloids of the plant *Sophora alopecuroides* [38] and for iridoid glycoside of the *Folium syringae* leaves [54]. Other indole alkaloids also show anti-inflammatory activity through the inhibition of NFκB [25,41]. This is unprecedented data on the protective activity of CLP in tissue damage in the model of ulcerative colitis.

Based on the results obtained in the present study, it was observed that two doses (40 and 4 mg/kg) of CLP were successful in reducing leukocyte recruitment in the zymosan-induced peritonitis model. When these two doses were applied to the therapy in the 3% DSS-induced ulcerative colitis model, the results showed that treatment with the 4 mg/kg dose of CLP presented promising results. This dose was able to attenuate weight loss and clinical signs, reduce colon size, decrease the levels of Th1 and Th17 pro-inflammatory cytokines and the expression of NFκB p65, and increase IL-10, which has anti-inflammatory activity. Therefore, the data presented in this study show the anti-inflammatory potential of CLP in the murine model of ulcerative colitis induced by DSS. Although we have shown that a possible mechanism of inhibition of the anti-inflammatory activity of caulerpin would be through inhibition of the signaling pathway of NFκB, which is a potent pro-inflammatory signaling pathway, we do not deny the possibility of CLP acting through other pathways, such as inhibiting the pathway of MAP kinases [41], through antioxidant activity [20] or increasing A2AR expression [31], and this last pathway plays an important role in the colitis [55].

## 4. Materials and Methods

### 4.1. Extraction and Purification of Caulerpin (CLP)

Samples of *Caulerpa racemosa* seaweeds were collected during the tides of syzygy (−0.2 to 2.0) in the coastal city Pitimbu, Paraiba, Northeast Brazil, coordinates 7007′31′′ S; 34049′25′′ W. The species were collected on 21 April 2013 and identified by Prof. Dr. George Emmamuel Cavalcanti de Miranda, Department of Molecular Biology in the Federal University of Paraiba (UFPB), and a voucher specimen was deposited in the Herbarium of Prof. Lauro Pires Xavier/UFPB under the number JPB 62814. The method chosen for the isolation of CLP was cold extraction with ethanol. The fresh algae material (dry weight, 5.0 kg) was extracted by thorough maceration (3×) with 95% ethanol (Merck, Darmstadt, Germany) at ambient temperature to obtain the ethanolic fluid extract. This extract was filtered and concentrated in vacuo under reduced pressure (T < 60 °C) to obtain the crude ethanolic extract (800 g). This crude extract was then dissolved in a hydromethanol solution (MeOH: H_2_O, 7:3, *v*/*v*) and subjected to a liquid–liquid partitioning process using the solvents hexane (Merck), dichloromethane (Merck), and ethyl acetate (Merck) to produce the hexane (82 g) and ethyl acetate (300 g) phases, respectively. The dichloromethane phase formed a precipitate which, upon recrystallization from acetone, produced a substance with orange red crystalline characteristics. This substance after identification by ^1^H and ^13^C NMR spectroscopic methods and comparison with literature data was identified as caulerpin (5 g, 0.1% of *C. racemosa* seaweed dry weight). Caulerpin is not a hygroscopic powder and is stable at room temperature and in the presence of light; it was kept under such conditions and stored in a clear glass vial throughout the experimental procedure. The whole process of extraction and purification was performed in the Laboratory of Pharmaceutical Technology/UFPB. Figure 1 shows the chemical structure of CLP [24].

### 4.2. NMR Spectra of Caulerpin

NMR spectra of CLP were obtained on a Bruker-200 MHz spectrometer operating at 200 MHz for ^1^H NMR and 50 MHz for ^13^C NMR. Deuterated chloroform (CHCl_3_) from Cambridge Isotope Laboratories (Tewksbury, MA, USA) containing TMS as an internal standard was used to obtain the experiments. ^1^H NMR (200 MHz, CDCl_3_) δ 9.21 (NH, *s*, 1H), 8.06 (H-9/9, *s*, 1H), 7.43 (H-4/4, *dl*, 1H), 7.31 (H-7/7′, *dl*, 1H), 7.20 (H-6/6′, *dt*, 1H), 7.12 (H-5/5′, *dt*, 1H), 3.90 (H-11/11′, *s*, 3H). ^13^C NMR (200 MHz, CDCl_3_) δ 166.8 (C-10/10′), 142.9 (C-9/9′), 137.8 (C-7a/7a′), 133.0 (C-2/2′), 128.3 (C-3a/3a′), 125.6 (C-8/8′), 123.5 (C-6/6′), 120.9 (C-5/5′), 118.4 (C-4/4′), 112.6 (C-3/3′), 111.7 (C-7/7′), 52.7 (C-11/11′).

### 4.3. Animals

Six- to eight-week-old male C57BL/6 mice were used in this study. They were kept under a light/dark 12-h light cycle, with free access to food and water. Three independent experiments were performed and each group comprised five animals. All procedures performed in this study were approved by the Experimental Animal Ethics Committee of the Biosciences Center of Federal University of Rio Grande do Norte (UFRN), under protocol number 007/2013.

### 4.4. Treatments

Treatment groups received CLP by oral route. For administration, CLP was suspended in a 5% gum Arabic (Synth, São Paulo, Brazil) solution (*w*/*v*) using the surfactant Tween 80^®^ (Sigma-Aldrich, Saint Louis, MO, USA). Each animal was treated orally using a gavage needle with a final volume of 200 µL for each treatment dose. The animals from control groups (vehicle and vehicle + zymosan or vehicle + 3% DSS) received by gavage 5% gum Arabic solution, as vehicle. In the peritonitis model, CLP was administered at the doses of 40, 4, and 0.4 mg/kg, after 30 min of zymosan (Sigma-Aldrich) inoculation. In the colitis model, the treatment with CLP at the doses 40 and 4 mg/kg was performed on alternate days. As glucocorticoids are the therapy of choice for the treatment of IBD [56], dexamethasone (Aché, São Paulo, Brazil) was used in the present study at a dose of 3 mg/kg to evaluate the efficacy of the CLP compared to the treatment currently in use. The dexamethasone solution was administered orally diluted in sterile saline 0.85%. 

### 4.5. Peritonitis Model

To evaluate the best dose of CLP that could have anti-inflammatory activity in vivo, we performed a zymosan-induced peritonitis model. Peritonitis was induced by intraperitoneal injection of zymosan at the concentration of 40 mg/kg. Thirty minutes after zymosan inoculation, the animals were orally treated with the vehicle or with three different doses of CLP (40, 4, and 0.4 mg/kg) or dexamethasone (3 mg/kg). Six hours after the zymosan injection, the mice were euthanized and a peritoneal cavity lavage was performed by the injection and aspiration of 5 mL of 0.85% saline solution. The obtained liquid was transferred into 15 mL tubes and centrifuged at 250× *g* for 10 min. The resulting cell pellet was resuspended in 1 mL of 0.85% saline solution. Since the inflammatory infiltrate found in the peritoneal exudate after 6 h of zymosan injection is known to be polymorphonuclear neutrophils [57], only the global leukocyte count was determined using the Neubauer chamber and dilution in Turk’s solution.

### 4.6. Colitis Model

Experimental colitis was induced by administration of dextran sulfate sodium (DSS) (molecular weight 36–50 kDa, MP Biomedicals, Santa Ana, CA, USA) diluted in drinking water for mice at a final concentration of 3% (*w*/*v*) for 7 consecutive days. The mice were orally treated with different doses of CLP one hour prior to the release of access to water containing 3% DSS (CLP 40 mg/kg or CLP 4 mg/kg + 3% DSS). Subsequent treatments were performed on alternate days (3rd, 5th, and 7th days) with the doses of 40 and 4 mg/kg of CLP, chosen on the basis of the peritonitis experiment, and the doses that best inhibited leukocyte migration. As a control drug, dexamethasone was used at a dose of 3 mg/kg (DEXA 3 mg/kg + 3% DSS). Negative and positive control groups were treated with the vehicle only; however, the positive control group received DSS (vehicle + 3% DSS) in the drinking water and the negative control group received water without DSS (vehicle + water). On the eighth day, the DSS solution was removed and replaced by water and on the ninth day the animals were euthanized. The liquid consumption was monitored daily to ensure that each group was consuming equivalent amounts of water. The development of colitis was assessed by the disease activity index (DAI), which considers animal weight loss, presence of blood in the stool, and stool consistency. The score of disease severity (Table 1) was determined and observed daily from the first day of DSS administration [58]. On the ninth day, the animals were euthanized. The colon was removed and measured. Subsequently, the colon was opened longitudinally and two fragments were extracted, one from the transverse and the other from the distal colon, which were extensively washed with a gentamicin (Neo Química, São Paulo, Brazil)/saline 0.85% (1/1000) solution for removal of all residual feces. Fragments of 1 cm of the distal colon were immediately fixed in formaldehyde solution 10% in sodium phosphate buffer (PBS) and used for histological analysis. Fragments of 0.5 cm of the transverse colon were used to perform tissue culture and subsequent dosage of cytokines. Prior to culturing, the transverse colon was extensively washed with culture medium RPMI-1640 (Gibco, Thermo Fisher Scientific, Waltham, MA, USA) containing 2% fetal bovine serum (Gibco) and gentamicin (1/1000) to remove any residual feces. Following the washing step, the tissue was deposited in a 24-well plate containing RPMI medium supplemented with 10% fetal bovine serum and gentamicin (1/1000). The plate was incubated for 24 h in a humidified chamber containing 5% CO_2_ at 37 °C. After incubation, the supernatants were collected and frozen for subsequent measurement of TNF-α, IFN-γ, IL-17A, IL-6, IL-10, and TGF-β1 levels by enzyme-linked immunosorbent assay kits (eBioscience, Thermo Fisher Scientific, and R&D Systems, Minneapolis, MN, USA) according to the manufacturer’s recommendations. For microscopic analysis, the distal colon fragments were arranged in a spiral shape [59]. The fixed material was sectioned, stained with hematoxylin/eosin (HE), and subsequently examined by light microscopy (Olympus, Tokyo, Japan) by a pathologist who was blinded to the group distribution.

### 4.7. Expression Analysis of NF-κB p65 Subunit

The total RNA from colon tissue (1 cm) of the mice was preserved in TRIzol^®^ (Sigma-Aldrich) for evaluation of the effect of treatment with CLP on the expression of NF-κB in a DSS-induced colitis model. RNA extraction was performed using the SV Total RNA Isolation System Kit (Promega, Fitchburg, WV, USA), following the manufacturer’s instructions. The cDNA synthesis from the extracted material was performed using the High Capacity cDNA Reverse Transcription kit (Applied Biosystems, Thermo Fisher Scientific, Foster City, CA, USA), following the manufacturer’s instructions. All material was stored at −20 °C for further analysis. Quantitative analysis of NF-κB p65 expression was performed by qPCR using the Fast SYBR Green^®^ Master Mix system (Applied Biosystems, Thermo Fisher Scientific, Foster City, CA, USA). Specific primers for the p65 subunit of NF-κB (F: 5′GCTCCTAAGGTGCTGACA3′; R: 5′ACCTCCGAAAGGAGATA3′, Integrated DNA Technologies Inc., Coralville, IA, USA) and for β-actin (F: 5′AGGCCAACCTGTAAAAGATG3′; R: 5′TGTGGTACGAGAGGCATAC3′, Bio Basic Inc., Markham, ON, Canada), which was used as the reference gene for normalization and comparative analysis of NF-κB p65 expression levels. The relative expression was calculated by the formula 2-ΔΔCT.

### 4.8. Statistical Analysis

The results were expressed as mean and standard deviations. The Mann–Whitney test was used to compare water + vehicle group and zymosan + vehicle or DSS + vehicle group. The one-way ANOVA test, with Tukey or Dunnet post hoc tests, was used to verify the presence of differences between the vehicle + 3% DSS group and the treatment groups. Tukey or Dunnet post hoc tests were performed for comparison of group means or to compare the means between specific groups. All statistical analyses were performed with GraphPad Prism 5.0 software (GraphPad Software Inc., San Diego, CA, USA). A *p* value of <0.05 was considered statistically significant.

## 5. Conclusions

In conclusion, our study revealed that treatment with CLP significantly reduced cell migration in the peritonitis model and ameliorated the clinical signs, histopathologic damage, and the production of pro-inflammatory cytokines in the colonic tissue in the colitis model, which is possibly linked with the inhibition of the activation of the NF-κB pathway. Therefore, these data strongly suggest that CLP could be a promising therapeutic medicine in the treatment of peritonitis and colitis. To clarify the exact target of CLP as well as further molecular mechanisms, more work should be done.

## Figures and Tables

**Table 1:** Score of disease severity [58].

Score	Weight Loss (%)	Rectal Bleeding	Stool Consistency
0	0–2	Absent	Well formed
1	>2–5	-	-
2	>5–10	Visible	Softened
3	>10–15	-	-
4	>15–20	Intense bleeding	Diarrheic
